# The microbiome shifts throughout the gastrointestinal tract of Bradford cattle in the Pampa biome

**DOI:** 10.1371/journal.pone.0279386

**Published:** 2022-12-20

**Authors:** Anderson Santos de Freitas, Flávia Caroline Gan, Diego Bittencourt de David, Luiz Fernando Wurdig Roesch

**Affiliations:** 1 Centro de Energia Nuclear na Agricultura, Universidade de São Paulo, Piracicaba, São Paulo, Brazil; 2 Centro Interdisciplinar de Pesquisas em Biotecnologia–CIP-Biotec, Campus São Gabriel, Universidade Federal do Pampa, São Gabriel, Rio Grande do Sul, Brazil; 3 Departamento de Diagnóstico e Pesquisa Agropecuária–DDPA, Secretaria Estadual da Agricultura, Pecuária e Desenvolvimento Rural–SEADPR/RS, São Gabriel, Rio Grande do Sul, Brazil; 4 Department of Microbiology and Cell Science, University of Florida, Gainesville, Florida, United States of America; The University of Sydney, AUSTRALIA

## Abstract

A deep understanding of the cattle gastrointestinal microbiome is crucial to selective breeding high-efficiency animals that produce more and generate less environmental damage. Here we performed the taxonomic identification of Bacterial and Archaeal communities using high throughput 16SrRNA gene sequencing from critical compartments of the gastrointestinal tract of Bradford cattle raised in a natural grassland in the Pampa biome, Brazil. We analyzed 110 samples, including saliva, ruminal fluid, and feces from 36 months old Bradford heifers (weighing on average 343 ± 30 kg by the sampling time). To reduce unexpected variation and confounders, we selected the animals from the same breed, submitted them to the same food source, and collected the samples for three consecutive years from different animals in the same season. Our main goal was to analyze the microbial shifts throughout the gastrointestinal tract to reference future works proposing management strategies and interventions to improve animal nutrition and increase production in the Pampa Biome. To accomplish our objective, we accessed the microbial community differences in groups with a high and low weight gain controlling for food ingestion and quality of grazed pasture. Few taxa were shared among the samples. About 40% of the phyla and 60% of the genera were unique from saliva samples, and 12.4% of the microbial genera were uniquely found in feces. All samples shared only 36.1% of phyla and 7.5% of genera. Differences in microbial diversity and taxa counts were observed. The ruminal fluid presented the lowest microbial richness, while saliva and feces presented the highest microbial richness. On the other hand, saliva and feces also presented more distinct communities between themselves when compared with ruminal samples. Our data showed that the saliva microbiome is not representative of the rumen microbiome and should not be used as an easy-to-collect sample for studies about the rumen microbiome.

## Introduction

Cattle breeding is a historically successful economic activity all over the world. Production of meat, milk, and leather has been present in humanity’s history since Classical Antiquity generating business and income for millions of people. Nowadays, the worldwide demand for food and animal sources is the leading reason scientists and producers have studied ways to improve chain production aiming for higher production and, as possible, fewer outlays [1, 2].

The Brazilian Pampa is a grassland-dominated environment characterized by an intense agriculture activity based on livestock in association with crop rotation using rice, soybeans, corn, and wheat [3]. These natural grasslands are dominated by about 450 gramineous species and nearly 200 leguminous species. Such characteristics of the Biome give it a natural aptitude for raising ruminants, an activity introduced in the 17th century. Nevertheless, the natural grasslands from the Brazilian Pampa present high seasonality of forage production and nutritional quality throughout the year. Management strategies aiming the rumen development and the establishment of rumen microbiota and interventions to improve animal nutrition should be implemented to increase production. Interventions into the cattle microbiome might be one of those strategies. For instance, using the probiotic bacteria *Bacillus subtilis natto* benefits the development of cellulolytic microbes in the gastrointestinal tract of calves [4]. Members of the genus *Coprococcus* have been associated with an efficient rumen microbiome capable of exhibiting increased propionate and butyrate production [5]. A stable microbial load of Lactobacillus species has been shown to improve weight gain and immunocompetence in young calves [6]. Still, a deeper understanding of the microbiome shifts along the gastrointestinal tract is necessary to take full advantage of these strategies.

The relationship between nutrient utilization and efficiency, the gastrointestinal microbiome, and animal nutrition is one of the leading topics in cattle breeding studies. Microbes along the bovine’s gastrointestinal tract are responsible for processing complex carbohydrates (cellulose and lignin), absorption of nutrients, and production of proteins [7]. The digestion starts in the mouth, where saliva enzymes first break down polymers into lower molecules while buffering substances keep the pH stable [8]. Bacteria from the *Proteobacteria*, *Tenericutes*, *Firmicutes*, and *Actinobacteria* phylum are the main taxa in the cattle mouth responsible for a healthy and stable environment [9]. Posteriorly, in the rumen and barely in the omasum, the bolus is fermented and produces volatile fatty acids (VFA), the primary food source for these animals. The rumen is composed of a diverse and stable community that includes Bacteria, Fungi, Archaea, and microeukaryotes [10–12]. Finally, the cud moves to the intestines after the rumination cycle, where non-previously degraded material is processed and absorbed through villi. Different and specialized bacteria such as *Plantomyces* and *Cloroflexi* are characteristic of this environment [13].

The knowledge about interactions between microbes has exponentially risen in the last decades with the Next-Generation Sequencing (NGS) development. This technique makes it possible to map the microbes in a biological sample without needing cultivation [14, 15]. Many studies were conducted, and many valuable findings were made in the rumen, such as the postulation of the core ruminal microbiota [12, 16], the prospection of probiotics to increase milk quality [17], and the identification of putative microbial enhancers of feed quality [18]. Nonetheless, many questions remain about how microorganisms act and respond in association with cattle, and new ones rise with any further report. For instance, we still need to know how the cattle microbiome can be effectively included as a trait in selection decisions and breeding programs or the relative contribution of environmental factors in determining the cattle’s microbiome composition.

Here we performed the taxonomic identification of Bacterial and Archaeal communities from critical compartments of the gastrointestinal tract of grass feed Bradford cattle raised in natural grassland in south Brazil. We hypothesized that different gastrointestinal compartments (saliva, ruminal fluid, and feces) harbor unique microbial communities. To test this hypothesis, we analyzed 110 samples from animals from the same breed, submitted them to the same food source, and collected them during the same season. Our main target was to characterize the microbiome shifts along the gastrointestinal tract to support future works aiming at generating interventions to improve rumen development, the establishment of a healthy rumen microbiota, and maintaining and improving animal nutrition.

## Materials & methods

### Experimental design

We collected samples from animals raised by the Departamento de Diagnóstico e Pesquisa Agropecuária (DDPA, SEAPI-RS), located in São Gabriel, RS, Brazil, for three years (2017–2019). All sample collections were done during the autumn to minimize seasonal variation and extreme climate conditions [19]. The Animal Welfare Committee approved animal management and research procedures of the State Foundation of Agricultural Research (FEPAGRO), registered under the number 16/13. No aggressive method was used in herding, aiming not to stress the animals.

We studied sets of Bradford heifers (58 animals) randomly distributed in paddocks with natural grasslands of the Pampa Biome. To obtain representative samples that allow us to detect Bradford’s core microbiome, different animals were sampled in a 3-year experiment. The heifers were 36 months old and, by the time of sampling, weighed 343 ± 30 kg. Each animal was sampled only once. Seventeen animals were sampled in the first year of the experiment. A different group of 17 animals was sampled in the second year, and 24 animals were sampled in the third year. All animals were submitted to a continuous grazing stock method, with a forage allowance of 12 kg of dry matter/100 kg of live weight adjusted by adding or taking extra animals to the paddock as needed. We monitored the weight gain for 60 days, with the sampling occurring in the middle of this period (30 days). Based on this monitoring, we estimate the average daily weight gain (ADG) for each animal and proceed to downstream analysis. Diet aspects such as weight gain, crude protein intake, and organic matter digestibility were accompanied for 120 days after the sample collection.

The native pastures included *Eryngium horridum*, *Vernonia nudiflora*, *Erianthus angustifolius*, *Eupatorium bunifolium*, *Paspalum notatum*, *Eragrostis plana*, *Axonopus affinis*, *Paspalum umbrosum*, *Desmodium incanum*, and *Paspalum plicatulum*.

### Sampling strategy and measurement of descriptive variables

Samplings (n = 110) were always performed after the morning grazing cycle (approximately 9:00 AM and 12:00 AM). During this procedure, all animals were kept in a closed corral without food assessment. Saliva (n = 35) was collected with an individually sterilized syringe and stored in a 5mL tube on ice. Ruminal fluid (n = 17) was collected through an esophageal probe equipped with a vacuum system using a sterilized set of hoses and kitassato flasks as previously described [20, 21]. Fecal samples (n = 58) were divided into two sub-samples. One sub-sample was stored in 5mL tubes and kept on ice while transported to the laboratory, where samples were frozen at -80°C until DNA extraction. The other sub-sample was dried with forced air circulation at 55°C for 72 hours. After partially dried and grounded, feces were subjected to dry matter determination after drying at 105ºC for 12 hours [22]. Organic matter in feces was determined by flaring crude protein (CP) at 550°C, according to the Kjeldahl method [23]. The concentration of fecal CP in the organic matter (CPf:g/kg.OM) was used in the Eqs. (I), (II), and (III) are described below to estimate organic matter digestibility (OMD), protein concentration in the diet (PC), and daily protein consumption (CP Intake). The equations were explicitly constructed for native grasslands of Pampa Biome by using Rosa et al. methods [21]:

OMD = 0:942–38,619/fecalCP (I)

PC = 1.346 * (fecalCP) - 347.63 (II)

CPintake = 8.0728 * (fecalCP) - 347.38 (III)

### DNA extraction and amplification

Microbial DNA was extracted from 0.25g of each sample (n = 110) using DNeasy PowerSoil Kit® (QIAGEN, Hilden, Germany), following the manufacturer’s instructions. DNA purity and concentration were determined using a NanoVue^TM^ spectrophotometer (GE Healthcare, Chicago, IL, USA). All DNA samples were stored at -20°C for downstream analyses.

Amplification, sequencing, and data processing were performed as described previously [24], following the recommendations of the Brazilian Microbiome Project [25]. Briefly, the V4 region from the marker gene 16S rRNA was amplified using the 515F/806R primers to amplify bacteria and archaea simultaneously. PCR products were purified with the Agencourt AMPure XP Reagent kit (Beckman Coulter, USA). The final concentration of DNA in the PCR product was quantified using the Qubit Fluorometer kit (Invitrogen), following the manufacturer’s recommendations. Finally, the PCR products were combined into equimolar concentrations and were used for library preparation with the Ion One-Touch 2 System using the Ion PGM Template OT2 400 Kit (Thermo Fisher Scientific, Waltham, MA, USA). Sequencing was performed with Ion PGM Sequencing 400 on the Ion PGM System using 318 Chips v2. The Raw sequences were deposited in the Sequence Read Archive (SRA), BioProject ID PRJNA818310. The individual accession numbers associated with each sample are available in S1 Table.

### Sequencing and amplicon analysis

Raw barcoded sequences were split into individual files utilizing FASTX-Toolkit [26]. These files were imported into the R environment [27], where all analyses were carried out. We performed quality filtering and built an amplicon sequence variants (ASVs) table through the DADA2 package [28], followed by taxonomy inference using the last available SILVA database (ver. 138.1) [29]. The resulting table was converted into a phyloseq object for downstream analyses [30]. Data were rarefied by the minimum library size [31] and transformed by centered log-ratio to reflect the compositional nature of this type of data [32]. Our dataset presented low variance with no need for controlling or strata (S1 Appendix).

We calculated the Good’s coverage for all samples [33] and evaluated the microbial distribution of taxa, diversity of ASVs, and distribution of phyla and genera among the three sample sources. Alpha diversity was measured by using the rarefied dataset with the microbiome package with a function called *alpha* [34]. Boxplots summarizing the alpha diversity distribution were plotted by using ggplot2 R package [35] The significance of numerical evaluations of alpha diversity, and abundance of phyla was tested with the Kruskal-Wallis *post hoc* Dunn test (p ≤ 0.05) from base R using the functions *kruskal*.*test* and *dunn*.*test*. Beta diversity differences among samples were measured by PERMANOVA analysis (p ≤ 0.05) using the *adonis* function from the vegan package [36] and plotted in a PCoA plot using the Euclidean distance and the phyloseq package and the functions *ordinate* and *plot_ordination* [30]. The statistical power of the sample number per group was estimated using the micropower R-package based on the permutational multivariate analysis of variance (PERMANOVA) [37]. The PERMANOVA powers were calculated based on the weighted Jaccard distance. The effect sizes (ω2) were calculated using matrixes of 80% powers for 58 fecal samples, 35 saliva samples, and 17 ruminal fluid samples. The R markdown file showing the amplicon analysis is provided at https://github.com/FreitasAndy/BovinesGIT.

## Results

We carried out a study with 110 samples from different compartments of the GIT of Bradford heifers (36 months, 343 ± 30 kg) distributed in paddocks with natural grasslands of the Pampa Biome, analyzing sequencing output, crude protein intake, an average daily weight gain and organic matter digestibility. With 58 fecal samples, 35 saliva samples, and 17 ruminal fluid samples, we obtained an 80% power that allowed us to detect small to medium effect sizes (Feces ω2 = 0.000132, Saliva ω2 0.0167, and ruminal fluid ω2 0.32). This number of samples provided the statistical power to answer questions about how similar or different the microbiota is from each part of the cattle’s GIT. The size of the dataset provided variability to validate the data and produce more reliable conclusions. Data analysis showed that we worked with a very homogeneous group. That means the animals presented a similar food intake and digestion capacity. On the other hand, all groups showed a wide range of weight gain (Table 1).

**Table 1:** Description of cattle diet variables per compartment of the gastrointestinal tract.

Parameters	Saliva (n = 35)	Ruminal fluid (n = 17)	Feces (n = 58)	p-value
Average daily weight gain (ADG, gd^-1^)	245.38 ± 30.4	323.47 ± 43.3	279.96 ± 115.7	0.063
Organic Matter Digestion (OMD, %)	65.3 ± 2.7	63.0 ± 5.0	64.3 ± 3.7	0.078
Crude Protein Intake (CPI, gd^-1^)	106.65 ± 17.9	107.28 ± 17.5	107.28 ± 17.5	0.550

Variables were summarized as average ± SEM and compared using the ANOVA. g = grams; d = days.

After sequence quality control, we obtained 990,505 high-quality reads associated with ASVs (a mean of 8,923 reads per sample) and 11,922 ASVs (The ASV table and associated taxonomy is presented in S2 Table, the complementary metadata associated with this experiment are presented on S3 Table). The coverage was higher than 99% in all samples, meaning our sequencing depth was sufficient for adequately investigating the microbiota in all samples, allowing us to include all samples in the downstream analysis.

### All GIT compartments presented different communities, but microbial communities from the rumen were less diverse

**Fig 1:**
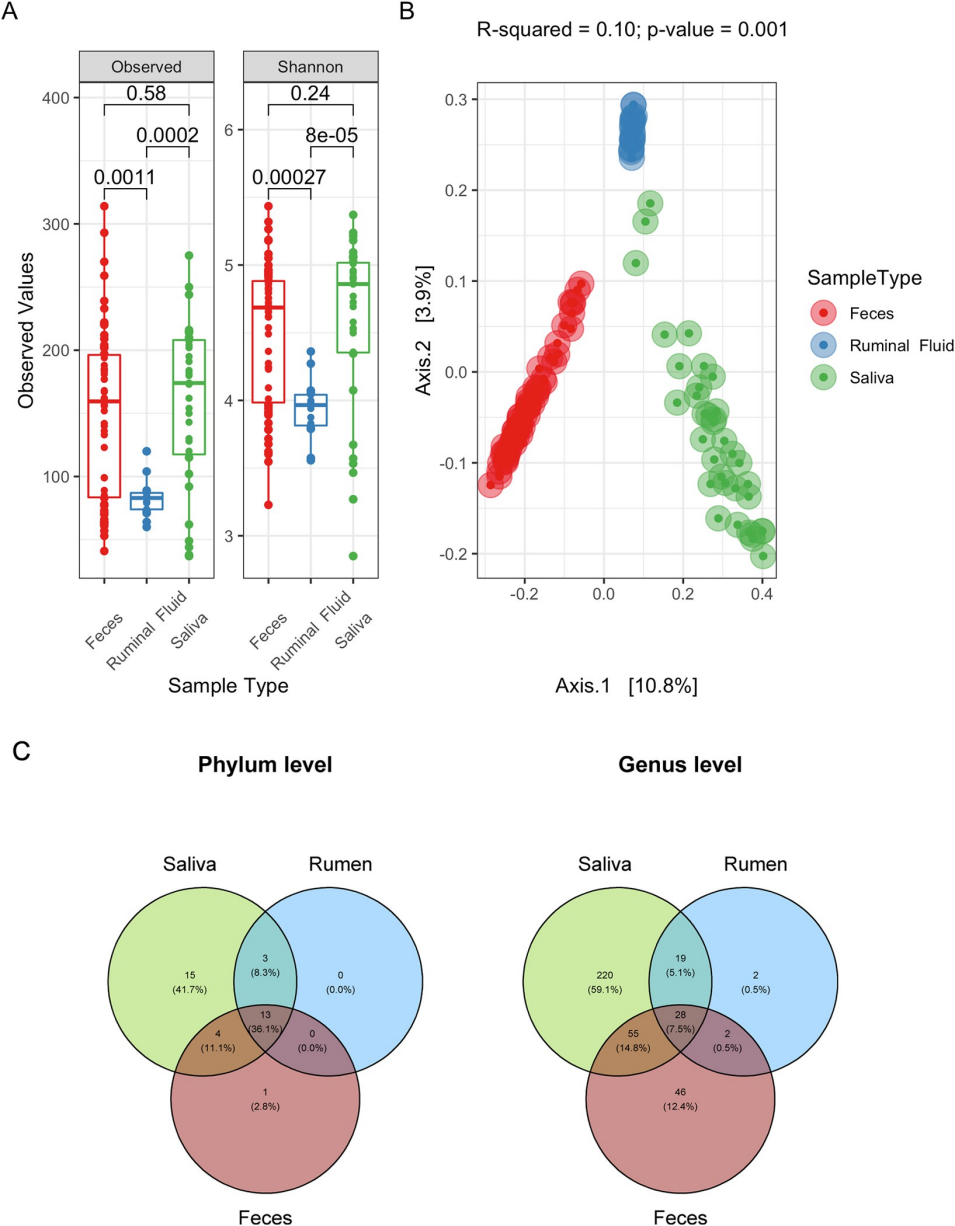
Differences in microbial communities among saliva (green, n = 35), ruminal fluid (blue, n = 17), and feces (red, n = 58). (A) The number of observed ASVs and Shannon diversity index in the three GIT sample sources. Boxes span the first to third quartiles; the horizontal line inside the boxes represents the median. Whiskers extending vertically from the boxes indicate variability outside the upper and lower quartiles. p-values were calculated with the t-test. (B) Bi-dimensional principal coordinate analysis (PCoA) based on Euclidean distance matrices comparing the microbial community of the three GIT sample sources. R^2^ and p-value were calculated by PERMANOVA analysis based on 999 permutations. (C) Venn diagram for the number of different phyla (left) and genera (right) shared within the three GIT sample sources. Numbers indicate how many taxa are unique from that source or shared with others.

Our goal was to measure the similarity among microbial communities from saliva, rumen, and feces. The ruminal fluid presented the lowest microbial richness and diversity (Fig 1A). Microbial communities from feces and saliva had similar richness and diversity. The beta diversity showed that all GIT sampling sources presented different microbial communities (Fig 1B, R^2^ = 0.1; p-value = 0.001). Although saliva and feces had similar microbial richness, they also had more distinct communities between themselves when compared with ruminal samples.

More than 40% of the phyla found in this study were unique from saliva samples (Fig 1C). No phyla were uniquely found in either feces or rumen. About 60% of the microbial genera found in our experiment were unique from saliva samples. On the other hand, 12.4% of the genera found in our investigation were uniquely found in feces samples. The rumen samples had less than 1% of unique sequences. All GIT sources shared only 36.1% of phyla and 7.5% of genera, confirming that each sampling source in the cattle’s GIT harbors a specialized microbial community.

### GIT sample sources have unique microbial communities and different microbial abundance

**Fig 2:**
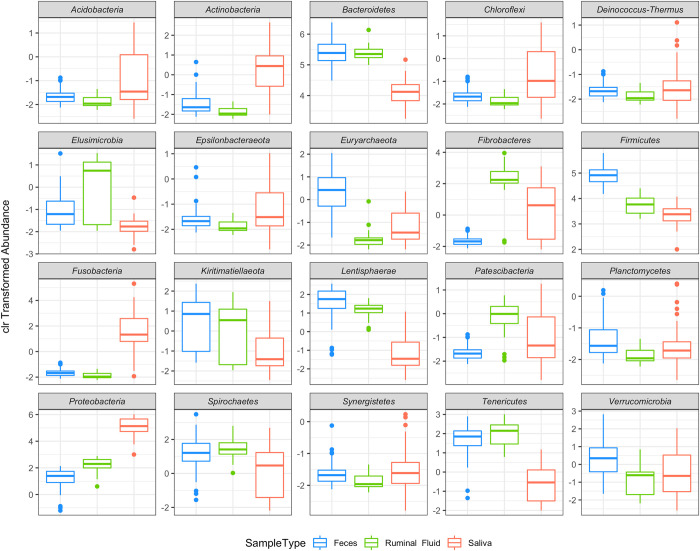
Centered log-ratio transformed abundance of the 20 more abundant microbial phyla present in saliva, ruminal fluid, and feces samples from beef cattle. Note that negative clr values indicate low microbial abundance.

No phyla presented the same abundance in the three GIT sources (S1 Table) (p-value ≤ 0.05). *Acidobacteria*, *Actinobacteria*, *Cloroflexi*, *Deinococcus-Thermus*, *Epsilonbacteraeota*, *Fusobacteria*, and *Synergistetes* presented decreased abundance in the rumen when compared with saliva (Fig 2). On the other hand, *Tenericutes* and *Fibrobacteres* presented lower abundance in saliva, higher abundance in the rumen, and lower abundance in feces (Fig 2). Besides, some phyla were negatively correlated. *Firmicutes* and *Proteobacteria*, for instance, presented a negative relationship irrespective of the GIT sampling site. This data showed that levels of *Firmicutes* increase over the bovines’ GIT, whereas *Proteobacteria* levels decrease over the GIT flow.

Other phyla were typically dominant in a specific GIT niche. *Bacteroidetes*, *Elusimicrobia*, *Kiritimatiellaeota*, *Lenthisphaerae*, *Planctomycetes*, and *Spirochaetes* presented lower abundance or were absent in saliva when compared to rumen and feces. Finally, *Verrucomicrobia* was the only phylum with a similar low abundance in saliva and rumen but more abundant in fecal samples.

**Fig 3:**
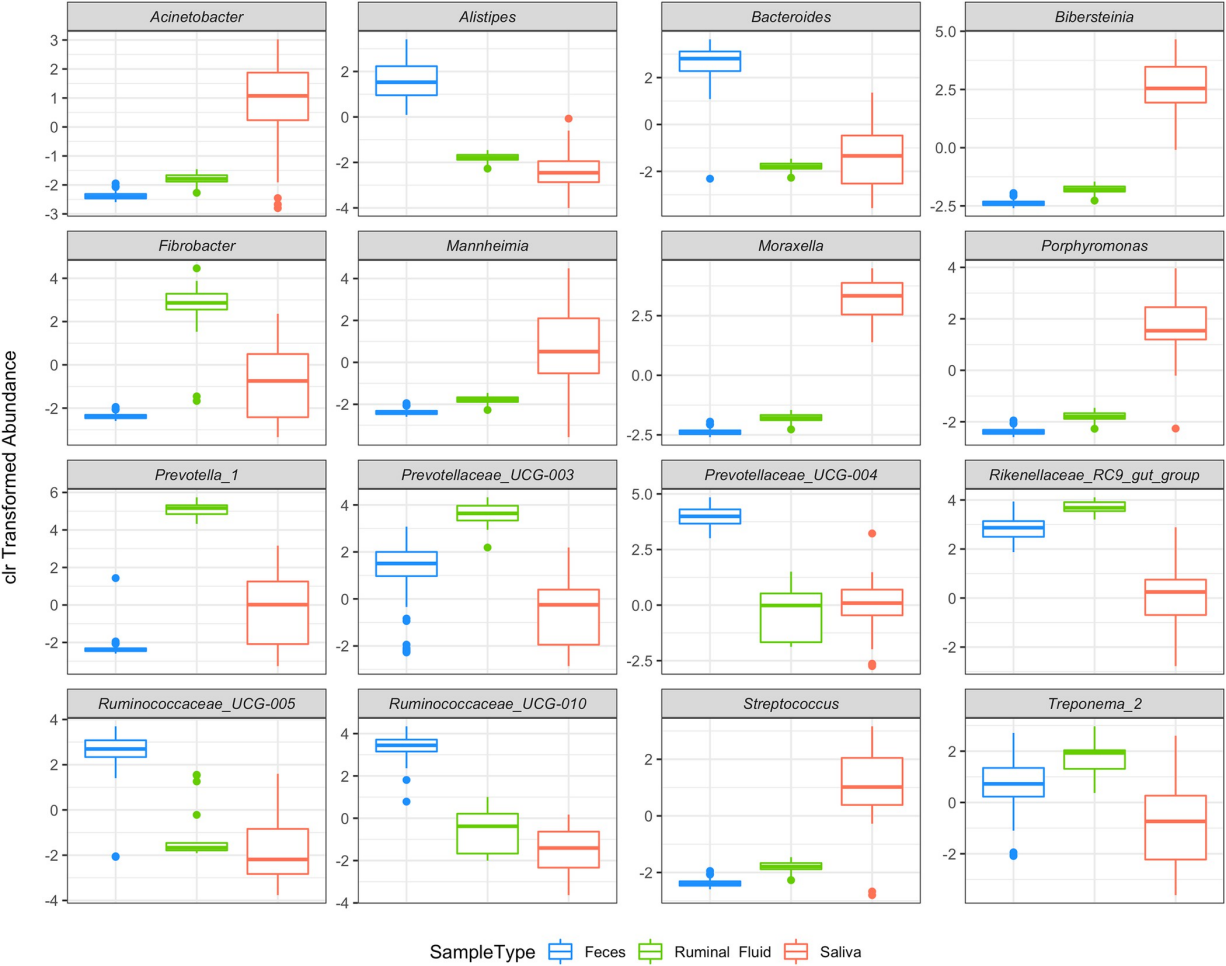
Centered log-ratio transformed abundance of the 16 more abundant microbial genera present in saliva, ruminal fluid, and feces samples from beef cattle. Note that negative clr values indicate low microbial abundance.

Important differences in the composition of the microbial community at the genera level were also observed among fecal samples, saliva, and ruminal fluid samples (Fig 3 and S5 Table). Among the most abundant genus, *Acinetobacter*, *Bibersteinia*, *Mannheimia*, *Moraxella*, *Porphyromonas*, and *Streptococcus* were enriched in saliva samples. On the other hand, feces samples were enriched by *Alistipes*, *Bacteroides*, *Prevotelaceae*_UCG-004, *Ruminococcaceae*_UCG-005 and *Ruminococcaceae*_UCG-010. *Fibrobacter*, *Prevoltella*_1, *Prevotellaceae*_UCG-003 *Rikenellaceae*_RC9_gut_group, and *Treponema*_2 were more abundant in the ruminal fluid samples.

### Animals with a higher weight gain do not ingest more food

We measured this on our samples by testing the relationship between the ADG and the crude protein intake (CPI), a known variable to assess diet quality, for each animal.

**Fig 4:**
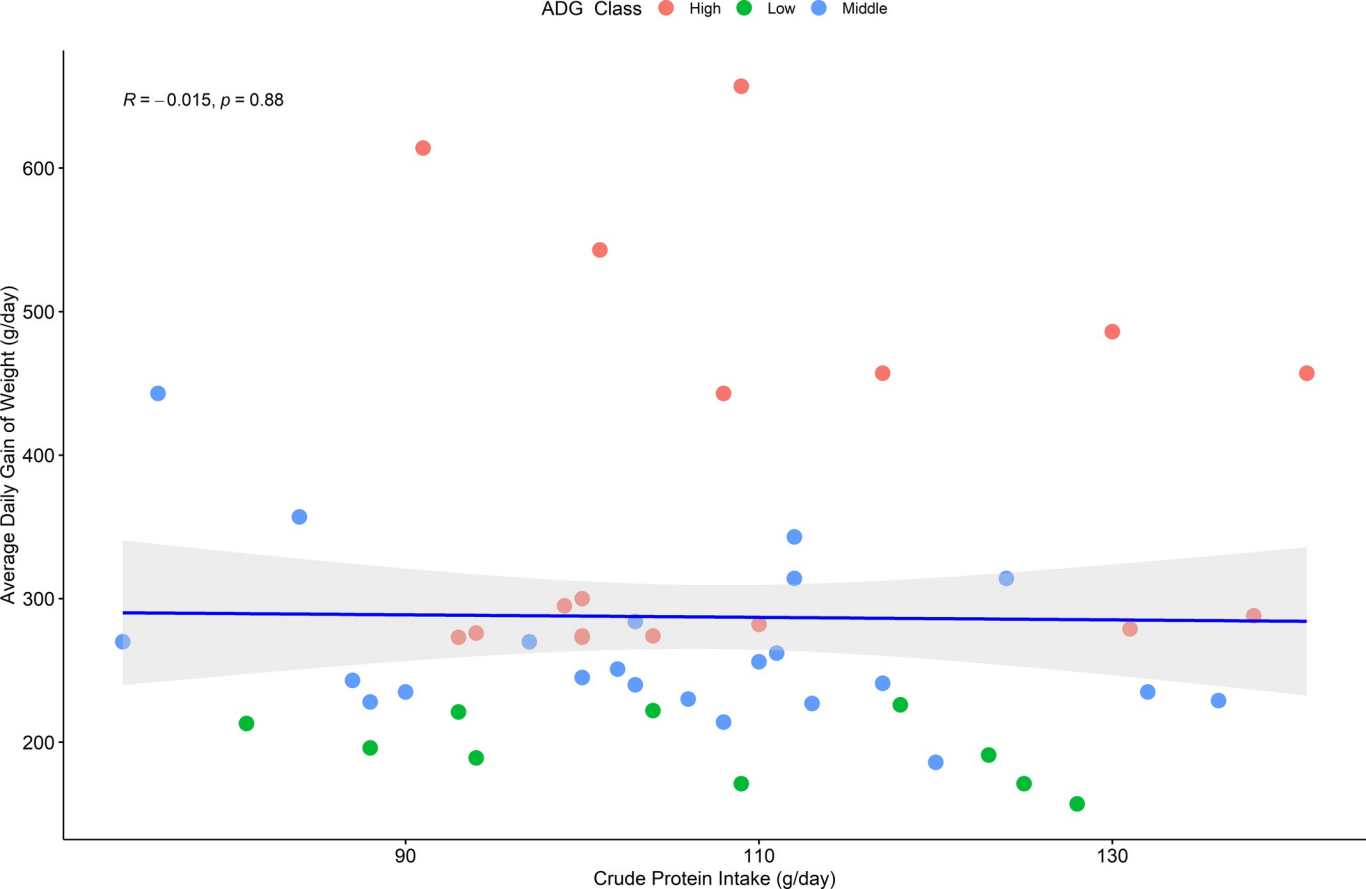
Spearman correlation between Average Daily Weight Gain (ADG) and Crude Protein Intake (CPI) in beef cattle (n = 110).

Our results showed no association between CPI and ADG. The CPI varied from 74 to 162 g day^-1^, but its variation does not follow any pattern compared with the ADG values from the same animals (p = 0.84, Fig 4).

## Discussion

Insights about how microbes correlate with cattle nutrition are crucial to selective breeding high-efficiency animals producing more and generating minor environmental damage [38]. Identifying a detailed cattle’s GIT microbiome profile will enable us to identify novel microbial markers, as well as their interactions with modifiable environmental factors, that are informative for establishing useful strategies aiming at interventions to improve animal nutrition. Here, we provide multiple lines of evidence that the microbiota changes throughout the GIT compartments despite the interaction between rumen and mouth via rumination.

Saliva, rumen, and feces samples collected from Bradford heifers (36 months old, 343 ± 30 kg at the sampling time) raised in natural grasslands of the Pampa Biome presented different microbiotas. Few taxa were shared between the different types of samples (Fig 1C). They also showed differences in weighted diversity (Fig 1B) and total taxa count (Fig 1A). Our findings contradict other works postulated that saliva samples could predict rumen microbes [39, 40]. Those papers pointed out that once ruminants pass considerable amounts of rumen material over their oral cavities by chewing the cud, oral samples may be good representations of the ruminal microbes. In this regard, targeting saliva instead of ruminal liquid might be valuable to rumen studies since the ruminal sample collection is complicated and invasive to animals [41]. Nonetheless, although these papers have shown valuable results in different aspects, both were conducted with few samples, presented low statistical confidence, and have not considered the compositional nature of their microbiome datasets [32]. Here, we provided a bigger sample size, more robust analysis methods, and deeper taxonomy resolution. Still, our results showed that the communities from different samples present different compositions (Fig 1). One of the reasons for that is the difference in many physiological aspects between the oral cavity and the rumen. Although the cud is shared between mouth and rumen, differences in pH and oxygen levels might be crucial factors contributing to these differences [8, 42]. Both compartments, in fact, share some taxa, but, saliva samples cannot resemble or predict ruminal microbes. For that reason, and based on our data, we do not contend this approach for future works once it could lead to misleading conclusions.

Differences in microbial diversity and the presence/absence of taxa in those sources were expected based on the physical and chemical nature of the GIT organs. Saliva is the first step of interaction between food and animal. It’s susceptible to airflow, produces buffering substances, and catalyzes fiber degradation keeping an alkaline pH between 8.5 and 8.9 [8, 43, 44]. The rumen is an anaerobic fermentative chamber that consumes 65 percent of the starch ingested by the animal, maintaining a barely acid pH of around 5.5 to 6.8 [8, 45, 46]. Feces are the end of the digestion after the bolus passes through the highly acidic abomasum (pH ~ 3) and turn into a more pleasant environment for microbes in the intestines [47–49]. Those differences make the rumen a more selective environment when compared with saliva and feces. Altogether these environmental restrictions lead to lower diversity (Fig 1A).

Saliva samples presented a more divergent community (Fig 1B and 1C), possibly because most saliva microbes cannot survive in the rumen environment, despite the high interaction between rumen and mouth via rumination [50]. Additionally, some ruminal taxa are expected to accompany the bolus through the intestines and remain in feces, which explains the closer beta diversity between them (Fig 1B). Also, the microbial diversity observed in feces samples indicates that the intestine is a more suitable environment for establishing microorganisms [51].

One interesting finding of our work was the negative association between the phyla *Firmicutes* and *Proteobacteria* among the different compartments of the GIT. Whereas the abundance of *Firmicutes* increases from the beginning to the end of the GIT, *Proteobacteria* does the opposite (Fig 2). A possible explanation for this is that *Firmicutes* can metabolize fat, which is degraded mainly in the small intestine, close to the end of digestion, where more specific phyla are needed for that [46]. The variation of *Proteobacteria* is less clear since the *Proteobacteria* phylum comprises a broad array of microbes that can perform multiple functions. Recent studies could not explain its variations along with the GIT [49].

Besides the phyla mentioned above, saliva samples were composed of *Acidobacteria*, *Actinobacteria*, *Cloroflexi*, and *Fusobacteria*. *Cloroflexi* and *Acidobacteria*. All are common phyla from the soil microbiome [52, 53], which could be ingested during grazing. *Fusobacteria* and *Actinobacteria* are responsible for states of healthy homeostasis in animals’ GIT and members of the saliva consensus microbiota [54, 55]. All these phyla are aerobic or microaerophilic. Probably the main reason why none or very few bacteria from these groups can survive in the anaerobic conditions of the rumen.

The rumen is a restrictive environment, with an increased abundance of phyla specialized in the anaerobic processing of organic matter. The fermentative capacity of this phyla might explain the high abundance of Bacteroidetes. The phyla include microbes from the genus *Prevotella*, one of the main bacterial genera in the rumen [12, 56]. Besides, the higher abundance of *Fibrobacteres*, part of the consensus ruminal microbiota, relies on the phylum’s importance for the degradation of fibers and complex carbohydrates such as cellulose [57].

Other prominent phyla in the rumen belonged to groups with a high capacity to adapt to stressful environments like the ruminal fluid. *Elusimicrobia*, *Kiritimatiellaeota*, *Lentisphaerae*, *Patescibacteria*, and *Tenericutes* are phyla of bacteria capable of growing in extreme conditions. Most of them are typically found at high depths in the ocean, in toxic wastes, and contaminated soils [58–62]. They found good conditions for survival in the ruminal environment as they can grow in anaerobic conditions [63].

The *Acidobacteria*, *Actinobacteria*, *Cloroflexi*, and *Fusobacteria* (Fig 2) presented a slightly higher abundance in fecal samples than in the ruminal fluid. These phyla are known to show less tolerance to anaerobic environments and belong to a group of the healthy intestinal microbiota [52–54]. On the other hand, *Deinococcus-Thermus*, *Epsilonbacteraeota*, and *Synergistetes* were detected in a similar abundance registered in saliva. The mechanisms behind this similar abundance are not fully understood. Still, somehow these phyla, which are present in saliva and the intestine of animals [64–66], can survive the passage through the rumen environment.

The main genera enhanced in saliva were *Moraxella*, *Porphyromonas*, and *Bibersteinia*, all of them gram-negative and oxidase-positive bacteria members of the regular oral cavity of animals [67, 68]. Those genera are aerotolerant anaerobes. They can tolerate, for a limited time, the presence of atmospheric oxygen, growing in the presence of oxygen concentrations in the mouth, allowing them to survive in saliva samples. On the other hand, in the rumen, there is an enrichment of bacterial genera which degrade complex carbohydrates, such as *Fibrobacter* and *Prevotellaceae* members [69, 70]. The pattern of anaerobic bacteria is also present in fecal samples, but with the increased abundance of *Bacteroides*, *Alistipes*, and *Ruminococcaceae* members, which are commonly found in animal and human feces but have less impact in the degradation of complex carbohydrates once these molecules are mainly degraded in previous compartments of cattle GIT [71].

Finally, the relationship between the ruminal microbiota and the ruminant’s performance apart from the diet isn’t novel [72]. Here we endorsed this concept by showing that Animals with a higher weight gain did not simply ingest more food (Fig 4). Salivary samples showed a clear microbial pattern in animals with a higher weight gain, relying on a higher abundance of microbial biomass degraders. Animals with a higher weight gain presented a higher abundance of *Lachnospiraceae XPB1014 group*, *Fibrobacter*, and *Prevotellaceae* members [57, 73, 74].

## Conclusions

This work hypothesized that different gastrointestinal compartments of the gastrointestinal tract (saliva, ruminal fluid, and feces) harbor unique microbial communities. The results obtained confirm our hypothesis. Few taxa were shared between the different compartments tested, and differences in microbial diversity and taxa counts were observed. Finally, our data showed that the saliva microbiome is not representative of the rumen microbiome and should not be used as an alternative, easy-to-collect sample for studies about the rumen microbiome.

## Supporting information

S1 Table Accession numbers associated with each sample deposited in SRA BioProject PRJNA818310. (XLSX)

S2 Table Amplicon Sequence Variant (ASV) table and associated taxonomy. (XLSX)

S3 Table Sample metadata associated with the experiment. (XLSX)

S4 Table Differences among the phylum abundance present in saliva, ruminal fluid, and feces from beef cattle after the Kruskal-Wallis post hoc Dunn test. The p-values ≤ 0.05 were considered significant.
(DOCX)

S5 Table Differences among the genus abundance present in saliva, ruminal fluid, and feces from beef cattle after the Kruskal-Wallis post hoc Dunn test. The p-values ≤ 0.05 were considered significant.
(DOCX)

S1 Appendix Distance-based test for homogeneity of multivariate dispersions. (DOCX)
