# Supplementary material for: The microbiome shifts throughout the gastrointestinal tract of Bradford cattle in the Pampa biome
Source: PLoS One. 2022 Dec 20;17(12):e0279386. doi: 10.1371/journal.pone.0279386 (PMC9767327; doi:10.1371/journal.pone.0279386)
Supplement: S4 Table — The p-values ≤ 0.05 were considered significant. (DOCX) [file pone.0279386.s004.docx]

**Table S1.** Differences among the phylum abundance present in saliva, ruminal fluid, and feces from beef cattle after the Kruskal-Wallis post hoc Dunn test. The p-values ≤ 0.05 were considered significant.

| Microbial Phylum | Statistic | p value |
| --- | --- | --- |
| *Acidobacteria* | 13.43670413 | 0.001208528 |
| *Actinobacteria* | 41.42059371 | 1.01E-09 |
| *Bacteroidetes* | 68.61736345 | 1.26E-15 |
| *Chloroflexi* | 20.15119554 | 4.21E-05 |
| *Deinococcus.Thermus* | 13.01369511 | 0.001493179 |
| *Elusimicrobia* | 16.02473304 | 0.00033134 |
| *Epsilonbacteraeota* | 8.319639174 | 0.015610374 |
| *Euryarchaeota* | 23.93760199 | 6.34E-06 |
| *Fibrobacteres* | 45.81093629 | 1.13E-10 |
| *Firmicutes* | 83.1354809 | 8.86E-19 |
| *Fusobacteria* | 59.51313403 | 1.19E-13 |
| *Kiritimatiellaeota* | 10.44066936 | 0.00540552 |
| *Lentisphaerae* | 53.55053897 | 2.35E-12 |
| *Patescibacteria* | 24.54921519 | 4.67E-06 |
| *Planctomycetes* | 6.262662177 | 0.043659644 |
| *Proteobacteria* | 80.74235862 | 2.93E-18 |
| *Spirochaetes* | 9.219332815 | 0.009955139 |
| *Synergistetes* | 7.798219681 | 0.020259938 |
| *Tenericutes* | 59.35486919 | 1.29E-13 |
| *Verrucomicrobia* | 9.467014155 | 0.00879557 |
