# Supplementary material for: The microbiome shifts throughout the gastrointestinal tract of Bradford cattle in the Pampa biome
Source: PLoS One. 2022 Dec 20;17(12):e0279386. doi: 10.1371/journal.pone.0279386 (PMC9767327; doi:10.1371/journal.pone.0279386)
Supplement: S5 Table — The p-values ≤ 0.05 were considered significant. (DOCX) [file pone.0279386.s005.docx]

**Table S2.** Differences among the genus abundance present in saliva, ruminal fluid, and feces from beef cattle after the Kruskal-Wallis post hoc Dunn test. The p-values ≤ 0.05 were considered significant.

| Genus | Statistic | p value |
| --- | --- | --- |
| *Acinetobacter* | 88.4031956 | 6.36E-20 |
| *Alistipes* | 63.8771268 | 1.35E-14 |
| *Bacteroides* | 58.2729748 | 2.22E-13 |
| *Bibersteinia* | 103.866906 | 2.79E-23 |
| *Fibrobacter* | 62.2648476 | 3.02E-14 |
| *Mannheimia* | 77.5149887 | 1.47E-17 |
| *Moraxella* | 103.864164 | 2.79E-23 |
| *Porphyromonas* | 99.896215 | 2.03E-22 |
| *Prevotella_1* | 74.029584 | 8.41E-17 |
| *Prevotellaceae_UCG.003* | 4.94585257 | 0.0843377 |
| *Prevotellaceae_UCG.004* | 5.27002903 | 0.07171793 |
| *Rikenellaceae_RC9_gut_group* | 18.260565 | 0.00010833 |
| *Ruminococcaceae_UCG.005* | 36.3278044 | 1.29E-08 |
| *Ruminococcaceae_UCG.010* | 3.72333034 | 0.15541362 |
| *Streptococcus* | 95.9984678 | 1.43E-21 |
| *Treponema_2* | 2.18825079 | 0.33483232 |
