# Supplementary material for: The microbiome shifts throughout the gastrointestinal tract of Bradford cattle in the Pampa biome
Source: PLoS One. 2022 Dec 20;17(12):e0279386. doi: 10.1371/journal.pone.0279386 (PMC9767327; doi:10.1371/journal.pone.0279386)
Supplement: S1 Appendix — (DOCX) [file pone.0279386.s006.docx]

Homogeneity of multivariate dispersions

Call: betadisper(d = ds, group = df$SampleType)

No. of Positive Eigenvalues: 109

No. of Negative Eigenvalues: 0

Average distance to median:

| Feces | Ruminal_Fluid | Saliva |
| --- | --- | --- |
| 26.98 | 25.60 | 26.83 |

Eigenvalues for PCoA axes:

(Showing 8 of 109 eigenvalues)

| PCoA1 | PCoA2 | PCoA3 | PCoA4 | PCoA5 | PCoA6 | PCoA7 | PCoA8 |
| --- | --- | --- | --- | --- | --- | --- | --- |
| 7690 | 2694 | 1555 | 1313 | 1164 | 1161 | 1123 | 1093 |

Analysis of Variance Table

Response: Distances

|  | Df | Sum Sq | Mean Sq | F value | Pr(>F) |
| --- | --- | --- | --- | --- | --- |
| Groups | 2 | 25.68 | 12.840 | 2.2794 | 0.1073 |
| Residuals | 107 | 602.73 | 5.633 |  |  |

ANOVA's p-value is not significant (Pr(>F) = 0.1073, meaning that group dispersions are homogenous.
